# 633. Nanopore Sequencing and In-house Bioinformatic Pipeline for Rapid and Accurate Detection of NRTI, NNRTI, PI, and INSTI Drug Resistance Mutations

**DOI:** 10.1093/ofid/ofad500.699

**Published:** 2023-11-27

**Authors:** Edsel Maurice Salvaña, Geraldine M Arevalo, Niña Theresa Dungca, Riacarl Alpay, Brian Schwem

**Affiliations:** Institute of Molecular Biology and Biotechnology, National Institutes of Health, University of the Philippines, Manila, National Capital Region, Philippines; University of the Philippines , Manila, National Capital Region, Philippines; Institute of Molecular Biology and Biotechnology, National Institutes of Health, University of the Philippines, Manila, National Capital Region, Philippines; Institute of Molecular Biology and Biotechnology, National Institutes of Health, University of the Philippines, Manila, National Capital Region, Philippines; University of the Philippines, Manila, National Capital Region, Philippines

## Abstract

**Background:**

Sanger-based sequencing (SBS) remains the gold standard for HIV-1 drug resistance testing (DRT). However, SBS output is still limited to an approximately 800-1000 bp read length. This is inadequate for amplifying the *pol* gene region that contains most of the clinically significant drug resistance mutation (DRM) positions, requiring separate steps for the protease (PR) and reverse transcriptase (RT) regions as is available in most commercial genotyping kits. Integrase (IN) gene is typically not sequenced, a major drawback since the WHO-recommended first-line treatment is now IN strand transfer inhibitor-based. We explore an alternative workflow for HIV-1 genotyping and DRT using the MinION sequencer to sequence a ∼2,800 bp amplicon covering PR and RT, and the first 224 codons of the IN region. This workflow also aims to reduce the turnaround time and cost of DRT.

**Methods:**

Thirty samples (VL 3,160-3,520,000 copies/mL) previously tested for drug resistance underwent PCR amplification using customized primers targeting the *pol* gene. Agarose gel electrophoresis and library preparation (SQK-RBK110.96 kit, Oxford Nanopore Technologies) were then performed, followed by MinION sequencing. An in-house bioinformatic pipeline was used to analyze the sequencing data.

**Figure:**
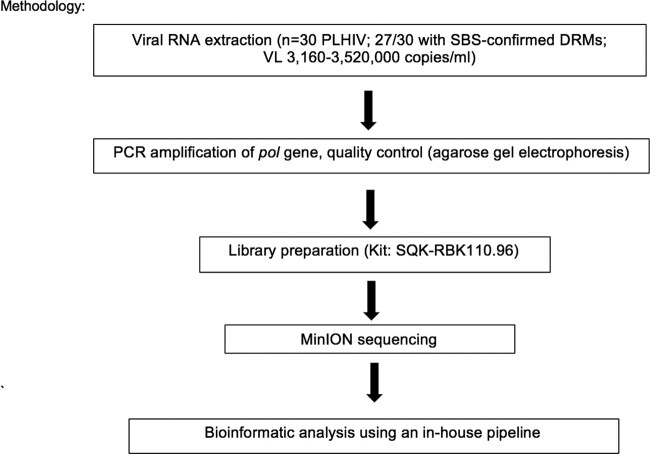

**Results:**

MinION sequencing was able to detect all DRMs detected by SBS in all 27 samples and did not detect any DRMs in the ones without DRMs on SBS. A few additional DRMs undetected by SBS were detected by MinION sequencing which is expected since MinION is known to have higher minority variant sensitivity. Comparison of the resistance profiles showed that SBS and MinION sequencing are concordant with a kappa value of 95.8 interpreted as having perfect agreement (Landis & Koch, 1977). DRMs from IN could not be properly evaluated due to lack of positive SBS controls but will be assessed at a later time using deep-sequenced controls. The entire workflow from RNA extraction to data analysis for all samples was completed within 22 hours, significantly shorter than that of the WHO protocol for HIV-1 DRT. The projected cost per run is comparable with that of SBS with a more rapid turnaround time.

**Figure:**
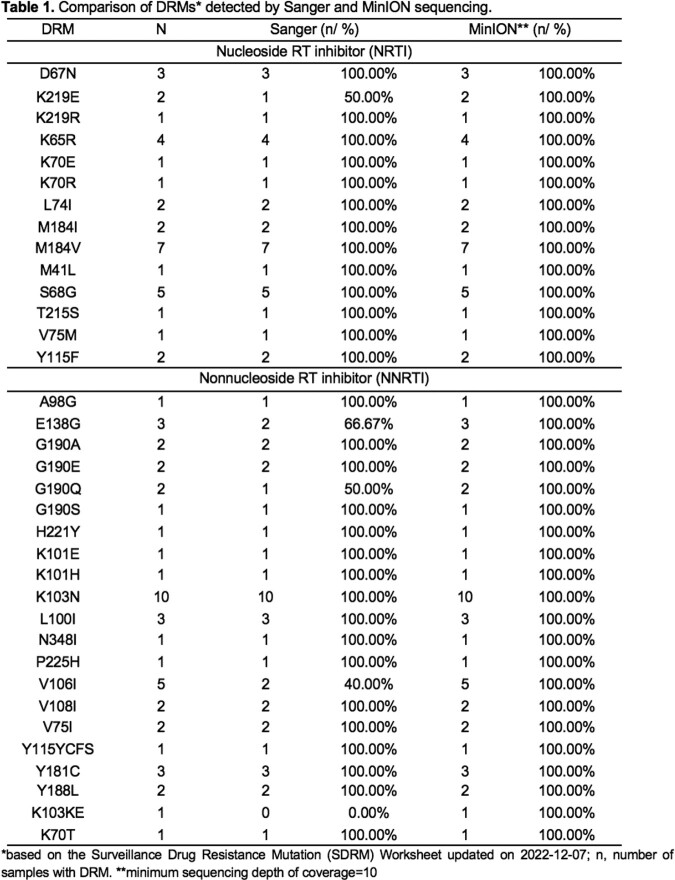

**Conclusion:**

Our findings show that MinION sequencing and our in-house analysis pipeline can rapidly and accurately detect DRMs in HIV-1 positive patients.

**Disclosures:**

**Edsel Maurice Salvaña, MD**, MSD: Advisor/Consultant|Pfizer: Advisor/Consultant

